# STEMIN and YAP5SA, the future of heart repair?

**DOI:** 10.3389/ebm.2024.10246

**Published:** 2024-10-31

**Authors:** Nada Bejar, Siyu Xiao, Dinakar Iyer, Azeez Muili, Adeniyi Adeleye, Bradley K. McConnell, Robert J. Schwartz

**Affiliations:** ^1^ Department of Biology and Biochemistry, University of Houston, Houston, TX, United States; ^2^ Department of Pharmacological and Pharmaceutical Sciences, College of Pharmacy, University of Houston, Houston, TX, United States

**Keywords:** stem cell factors, OCT3/4, SOX2, KLF4, and C-MYC (OKSM), serum response factor, STEMIN, YAP5SA

## Abstract

This review outlines some of the many approaches taken over a decade or more to repair damaged hearts. We showcase the recent breakthroughs in organ regeneration elicited by reprogramming factors OCT3/4, SOX2, KLF4, and C-MYC (OKSM). Transient OKSM transgene expression rejuvenated senescent organs in mice. OKSM transgenes also caused murine heart cell regeneration. A triplet alanine mutation of the N-terminus of Serum Response Factor’s MADS box SRF153(A3), termed STEMIN, and the YAP mutant, YAP5SA synergized and activated OKSM and NANOG in adult rat cardiac myocytes; thus, causing rapid nuclear proliferation and blocked myocyte differentiation. In addition, ATAC seq showed induced expression of growth factor genes *FGFs*, *BMPs*, *Notchs*, *IGFs, JAK, STATs* and non-canonical *Wnts.* Injected STEMIN and YAP5SA synthetic modifying mRNA (mmRNA) into infarcted adult mouse hearts, brought damaged hearts back to near normal contractility without severe fibrosis. Thus, STEMIN and YAP5SA mmRNA may exert additional regenerative potential than OKSM alone for treating heart diseases.

## Impact statement

The induction of reprograming factors, OCT3/4, SOX2, KLF4, and C-MYC (OKSM), truly stands out from a myriad of regeneration studies, for their rejuvenation of senescent organs, such as the adult heart. However, long term treatment of OKSM, as with adenoviral expression, elicited cancers. Short term transfections of a regenerative cocktail STEMIN, and YAP5SA synthetic mmRNA induced OKSM plus Nanog, and rejuvenated infarcted hearts. Short-term treatments with STEMIN and YAP5SA mmRNA delivery may become a safer strategy to treat debilitating human cardiac diseases.

## Introduction

We showcase breakthroughs in stem cell factor [1, 2] and STEMIN and YAP5SA [3, 4] in heart regeneration. Human adult heart lacks the intrinsic regenerative capacity to self-repair after cardiac injury, such as a myocardial infarction (MI). Many of the patients with ischemic heart disease not only undergo the acute phase of MI, but also develop ischemic cardiomyopathy, due to the loss of cardiomyocytes, and decreased cardiac function culminating in heart failure [5]. Due to the low regenerative capacity of cardiomyocytes, the damaged myocardium is replaced by fibrotic scar tissue, which further reduces pumping and circulatory function of the heart. Subsequently, the cardiac remodeling process results in further fibrosis, loss of cardiomyocytes, decrease cardiac function, and eventually resulting in heart failure, the leading cause of death worldwide [6].

Protecting the heart from progression to fatal heart failure continues to be focus of treating ischemic heart diseases [7, 8]. Cardiac intervention via revascularization by thrombolysis, and bypass surgeries to improve blood supply can salvage the injured ischemic myocardium. Medications such as angiotensin-converting enzyme inhibitors, angiotensin receptor-neprilysin inhibitors, mineralocorticoid-receptor antagonists, and β-blockers were proven to be effective on decreasing heart failure mortality [6, 9, 10]. Patients could benefit from these cardioprotective therapies targeting the remodeling process in the failing hearts. However, efficacious therapies for advanced cardiac remodeling in the later stages of heart failure are limited [11]. Mechanical support therapies such as cardiac resynchronization therapy and the application of left ventricular assist devices show beneficial contributions to end-stage heart failure patients [12], but the only treatment to end-stage heart failure with definitive effects is heart transplantation, which is limited by the lack of donor hearts [13].

Virtually the complete supply of human cardiomyocytes is established within the first month of life, and there is a dramatic drop in regenerative capacity within the first few days after birth [14]. Naqvi et al. [15] showed that the IGF-1/IGF-1-R/Akt pathway can be activated by a thyroid hormone surge in juvenile mice and initiated a brief but intense cardiomyocyte proliferative burst. Cardiomyocyte proliferation contributes to developmental heart growth in children. The number of cardiomyocytes in the left ventricle increased 3.4-fold between the first year and 20 years of age [16]. Adult human myocytes still maintain the ability to renew at approximately 1% per year, which was revealed by carbon-14 dating experiments [17]. Therefore, the poor regenerative capacity of adult human cardiomyocytes severely limits myocardial repair after a cardiac scenario. This review will survey potential therapies for the promotion of cardiomyocyte endogenous regenerative capacity towards cell replacement and cardiac repair.

## Cell cycle regulation

Cell cycle regulators were among the first factors reported to be sufficient for driving adult cardiomyocyte through cell cycle, long before the trans-differentiation methods were published. In 2004, CNNA2 was reported to induce cardiac enlargement by cardiomyocyte hyperplasia, when expressed from embryonic day 8 into adulthood [18]. Intramyocardial delivery of adenoviral vector expressing CNNA2 could induce myocardial regeneration and enhance cardiac function in injured heart [19] and constitutive expression of CNNA2 could limit ventricular dilation while enhancing cardiac function [20]. Besides CNNA2, other cyclins such as CNND1, CNND2, and CNND3 were also proved to promote cardiomyocyte cell cycle activity [21, 22]. A discrete combination of cell cycle regulators besides cyclins were reported to efficiently unlock the proliferative capacity in cardiomyocytes that have terminally exited the cell cycle. Overexpression of four factors cyclin-dependent kinase 1 (CDK1), CDK4, CNNB1, and CNND1 indicated as 4F could drive robust cell proliferation in post-mitotic mouse, rat, and human cardiomyocytes, whereas CDK1 and CNNB can be substituted by small molecules SB431542 and MK1775 [23].

## Growth factor stimulants

Growth factors were also described to have the ability to stimulate mature cardiomyocytes entry into cell cycle. FGF1/p38 MAP kinase inhibitor treatment after acute myocardial infarction in 8 to 10-week-old adult rat could increase cardiomyocyte mitosis. FGF1/p38 MAP kinase inhibitor treatment of 4 weeks resulted in reduced scar tissue and improved heart function [24]. However, a randomized clinic trial did not support the strategy of p38 MAPK inhibition in patients hospitalized with myocardial infarction. Losmapimod, a selective, reversible, competitive inhibitor of p38 MAPK, did not reduce the incidence of recurrent major adverse cardiovascular events in patients hospitalized with acute myocardial infarction [25]. In a swine model, IGF-1/HGF therapy was able to improve cardiac function in chronic myocardial infarction heart, and further increases can be observed by using an improved new delivery method, UPy hydrogel [26]. Nevertheless, treatments of growth factors not only stimulate the capacity of cardiomyocytes to re-enter cell cycle, but also fibroblasts to enter the cell cycle [26].

## Manipulate signaling pathways

Signaling pathways involved in cardiogenesis and cardiomyocyte maturation were also investigated for their ability to promote cardiomyocyte regeneration. Meis1 deletion in mouse cardiomyocytes was sufficient to extend the proliferative window of postnatal cardiomyocytes and reactivate cardiomyocyte mitosis in adult mouse heart without deleterious influences [27]. Paracrine factors such as Fgf16 were also reported to be potential regulatory factors in promoting myocardial repair [28]. GATA4 regulates neonatal heart regeneration through regulating expression of FGF16, and overexpression of FGF16 via adeno-associated virus in Gata4-ablated mice heart could partially rescue cardiac hypertrophy and improve cardiac function after injury. Tbx20 overexpression in adult cardiomyocyte directly represses cell-cycle inhibitory genes Meis1, Btg2, and p21, hence promotes adult cardiomyocyte proliferation and preserves cardiac function after myocardial infarction [29]. Hippo signaling pathway has appeared to be a key regulator of cardiomyocyte proliferation [30–33]. MicroRNAs such as miR302-367 cluster have been shown to regulate cardiomyocyte proliferation [34]. miR590 and miR199a were reported to act as key regulators of cardiomyocyte proliferation [35].

## Cell reprogramming

In the past decade, with the advent of iPSC technology, numerous cell differentiation methodologies have been developed [36–38]. Somatic cell reprogramming of adult murine cardiac fibroblasts into beating cardiac-like myocytes *in vitro* were first established by the introduction of four transcription factors, GATA4, HAND2, TBX5, and MEF2C [39]. Also, microRNAs were proven to mediate somatic cell transdifferentiation into cardiomyocyte-like cells. For example, a combination of microRNAs (miR-1, miR-133, miR-208, and miR-499) could induce direct cellular reprogramming of fibroblasts to cardiomyocyte-like cells both *in vitro* and *in vivo*. [40] The authors demonstrated that a single transient transfection of the miRNAs was able to mediate reprogramming confirmed by expression of mature cardiomyocyte markers, exhibition of cardiomyocyte spontaneous calcium flux characteristic, and sarcomeric organization. Wang et al. [40] demonstrated that the introduction of “GMT” factors Gata4, Mef2c, and Tbx5 could mediate the resident non-cardiomyocyte in the murine heart to be reprogrammed into cardiomyocyte-like cells *in vivo.* Islas *et al.* [41] reported that mammalian mesoderm posterior (MESP) homolog and v-ets erythroblastosis virus E26 oncogene homolog 2 (ETS2) can reprogram primary human dermal fibroblasts into cardiac progenitor cells, whereas Nam *et al.* [42] showed that four human cardiac transcription factors, GATA4, Hand2, T-box5, myocardin, and two microRNAs, miR-1 and miR-133, can activate cardiac specific marker expression in both neonatal and adult human fibroblasts. Purely chemical means by introduction of small molecules and chemical cocktails were soon discovered to conduct direct reprogramming of fibroblasts to functional cardiomyocytes. Treatment of a combination of nine compounds termed 9C to can reprogram human fibroblasts to uniformly contracting induced cardiomyocyte-like cells [43]. Bypassing the use of viral-derived factors, automatically beating cardiomyocyte-like cells could be generated from mouse fibroblasts only by addition of chemical cocktails instead of transcription factors [44]. The studies of purely chemical means replacing viral-derived factors laid foundations for potential safer treatment for heart failure.

## Reprograming factors, OKSM

Recently, short-term *in vivo* transgene induction of reprogramming factors OCT3/4, SOX2, KLF4, and C-MYC (OKSM) for less than a week generated partial reprograming, rejuvenated senescent organs, and extended mouse lifespans [1]. Transgenic expression of OSKM *in vivo* improves recovery from metabolic disease and muscle injury in older wild-type mice. Partial reprogramming may, lead to rejuvenating effects in different tissues, such as the kidney and skin [45]. The rejuvenating effects were associated with reduced expression of genes involved in inflammation, senescence and stress response pathways. Mechanistically, epigenetic chromatin remodeling occurs during shorter term OKSM treatment which coincides with anti-aging. But, long term transgene expression by adenoviruses may cause tumorgenesis [45].

Indeed, a recent study showed that *in vivo* expression of OKSM transgenes caused murine heart cell regeneration [2]. Short-term expression of OKSM did not cause cancer but was sufficient to induce cell replication and rejuvenation. However, long term treatment of OKSM, as with adenoviral expression, elicited cancer like transformation. Thus, to rejuvenate senescent myocytes and expand their number after a cardiac infarct, adult myocytes may need to be taken backwards to a primitive replicative state driven by stem cell factors for short term expression. Avoidance of long term expression from viral vectors provide a strong rationale of the use of synthetic mode RNA for short term transfections into cardiac myocytes.

## Synthetic RNA delivery to cardiac myocytes

The idea of gene transfer by mRNA as a method to transfer somatic genes into mammalian tissue was first introduced, by Bhargava and Shanmugam [46]. Wolff *et al.* [47] injected vectors expressing mRNA encoding luciferase, chloramphenicol acetyltransferase, and β-galactosidase into mouse skeletal muscle *in vivo*. Protein expression was detected for all the genes, which marked the opening for the use of mRNA as a method to somatic gene transfer method into mammalian tissue. However, this method had limited use because of the immune response that mRNA elicited [48]. Unmodified mRNAs can be recognized by the innate immune system of the cells via toll-like receptors [49], thus promoting the degradation of the unmodified mRNA. Fortunately, modified mRNA was made to bypass toll-like receptors. Modifying mRNA’s (mmRNA) secondary structure, substituting uridine with pseudouridine, and replacing cytosine with 5-methyl-cytosine can all lead to less recognition by nucleases and toll-like receptors [49].

## STEMIN and YAP5SA induced OKSM

**FIGURE 1:**
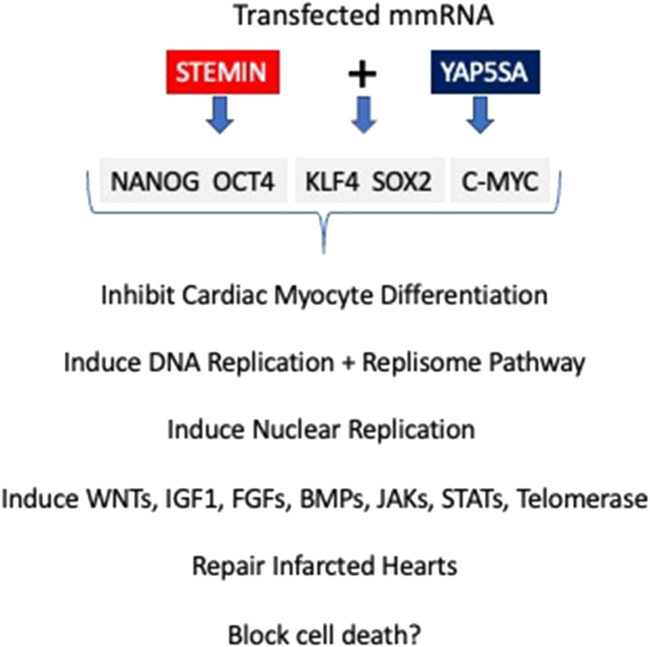
Schematic diagram of STEMIN and YAP5SA synthetic mmRNA induction of the cardiac myocyte regeneration pathway. STEMIN and YAP5SA synergize by the activation of the stem cell factors OCT4, KLF4,SOX2 and C-MYC (OKSM) + Nanog. Evidence provided by Chen et al. [2] and Xiao et al. [3, 4] showed that OKSM treatment of adult cardiac myocytes has a fundamental role in inducing replication and the inhibition of myocyte differentiation, taking cardiac myocytes backwards to a more primative developmental state. In addition, Xiao et al. [3, 4] showed that STEMIN and YAP5SA growth factor pathways plus telomerase maintence gene activities repaired infarcted mouse hearts and the potential for blocking cell death.

A schematic diagram of STEMIN and YAP5SA synthetic mmRNA induction of the cardiac myocyte regeneration pathway. STEMIN and YAP5SA synergize by the activation of the stem cell factors OCT4, KLF4,SOX2 and C-MYC (OKSM) + Nanog, shown in Figure 1. Evidence provided by Chen et al. [2] and Xiao et al. [3, 4] showed that OKSM treatment of adult cardiac myocytes has a fundamental role in inducing replication and the inhibition of myocyte differentiation, taking cardiac myocytes backwards to a more primative developmental state. Xiao et al. [3] discovered that a triplet alanine mutation of N-terminus of SRF’s MADS box SRF153 (A3), termed STEMIN, showed powerful activation of stem cell factors, and inhibited the induction of sarcomere assembly factors and cardiac myocyte specific genes. The triplet alanine mutation at aa153, aa154, and aa155 of the N-terminus of SRF’s MADS box blocked the interaction of Nkx2.5 and GATA4 required for facilitating SRF DNA binding to CArG boxes; thus, blocking myocyte differentiation. Xiao *et al.* [3] showed the ability for STEMIN to be the “myogenic driver” was completely abrogated in the SRF null ES cells. The mutation of aa154 lysine to an alanine in the MADS box severely weakened SRF153(A3) transcription of many CArG-dependent cardiac-specified genes. Rescue of SRF null ES cells with lentiviral expressed triplet SRF mutant, STEMIN inhibited the induction of several cardiac myocyte specific genes, such as those encoding sarcomeric actins, heavy and light chain myosins, ion channels, and structural proteins. And caused powerful activation of stem cell marker genes, such as *Egr1*, *Rex1*, *Nanog*, *Oct4*, *Zic3*, *Dppa2*, *Dnmt1*, *Dnmt2*, and *proliferin* [3].

## Constitutive YAP1 activity by mutant YAP5SA

Transcription co-activator YAP can be an effective target to manipulate due to its function, as the key regulator in Hippo signaling pathway. Zhao *et al.* [50] generated and active form of YAP, termed YAP5SA, by mutating all the LATS1/2 phosphorylation sites. The phosphorylation sites mutation of YAP prevents 14-3-3 binding, thus preventing YAP protein degradation. YAP5SA enters nucleus and binds with TEAD to regulate nuclear targets. Recently, YAP5SA has been proven to partially reprogram the highly differentiated adult mouse cardiomyocytes to a more primitive proliferative state [51].

The mutual role of STEMIN and YAP5SA synthetic mmRNA was tested in adult rodent cardiomyocytes. Xiao at al [3]. showed adult cardiomyocytes entered the mitotic cell cycle 24 h post-transfection. Their synthetic mmRNA declined by at least 90% within 24 h and was undetectable by 48 h supported the notion of the rapid turnover of mmRNA. We then asked, how does STEMIN and YAP5SA activate nuclear replication so quickly? Azeez Muili, a recent doctoral student, discovered that transfection of neonatal rat ventricular myocytes (NVRM) with STEMIN mmRNA for 24 h revealed the induction of NANOG by anti-NANOG staining, and significant induction of NANOG and OCT4 RNA, but not KLF4, SOX2 and C-MYC transcripts assayed by quantitative PCR and by RNA sequencing [3]. In fact, in comparison to transfected YAP5SA, NANOG, and OCT4 transcripts were induced to a greater extent with STEMIN, while YAP5SA upregulated C-MYC. Together STEMIN and YAP5SA synergized and induced KLF4 and SOX2 and the stem cell program similar to short term OKSM transgenic expression [2].

Next, the expression of cyclins appeared to be repressed in murine ES cells in the absence of SRF. Rescue with wild-type SRF caused activation of cyclins, CNNB1, CNND1, CNNC, and CNNE1, while STEMIN strongly induced CNNA2, CNNB1, and CNNE1. Note the induction of CNNA2 fostered myocardial regeneration and enhance cardiac function in injured heart [19, 20]. Most of the crucial genes involved in DNA replication in the replisome pathway, such as ORC2, MCM2, CDC45, and CLASPIN, were significantly increased by STEMIN and YAP5SA mmRNA in the G1 phase of the cell cycle. Mitotic genes such as, *Bub1*, *Bub1b*, *Cenpe*, *Ndc80*, *CcnB1*, and *Dync1* was observed by 32 h and the appearance of DNA packaging genes, which mark the S phase of the cell cycle, including histone 1 genes, such as *Hist1h1a*, *Hist1h1b*, and *Hist1h2ba*, by 40 h post transfection. Upregulation of crucial cell cycle genes such as Plk1 and Anln suggested that STEMIN and YAP5SA promoted several steps of cell-division cycle of cardiomyocyte. In addition, DIAPH3 was localized to multiple regions between and surrounding dividing nuclei [3, 4]. DIAPH3 marks anaphase of the cell cycle and induced F-actin to help assemble a contractile ring during cytokinesis. By 40 h post-STEMIN and YAP5SA treatment, many cardiac-specified genes including *Actc1*, *Myh6*, *Myocd*, and *Mef2C* were downregulated. Thus, STEMIN and YAP5SA mmRNA is a potent activator of stem cell gene activity of OKSM plus Nanog, cell replication and inhibitor of cardiac-specific gene activity.

A new molecular technology named ATAC seq (Assay for Transposase-Accessible Chromatin using sequencing) accesses remodeled open chromatin DNA with an hyperactive mutant Tn5 Transposase that inserts sequencing adapters into open regions of the genome [52]. Sequencing TnT5 bound DNA revealed regions of increased accessibility and maps transcription factor binding sites. To identify the underlying mechanism of how STEMIN works as a novel transcription factor, we used ATAC-seq to create a bioinformatics topography of interactomes of STEMIN, wildtype SRF, and YAP5SA. Xiao et al. [3] findings suggest a complementary effect of YAP5SA and STEMIN interactions with known and novel co-factors.

SRF has several tissue-specific regulatory cofactors, such as Nkx2.5 and GATA4, that control SRF activity by interacting with SRF’s MADS box [3]; whereas, YAP does not directly bind to DNA or bind directly to SRF [53]. ETS factors bind well to wildtype SRF as previously shown [54–56] and to mutant STEMIN [3]. TEAD1 or TEF1, one of SRF’s cofactors shown by our previous studies [57, 58] to physically interact with SRF, may also serve as a bridge between YAP5SA and STEMIN to implement their synergy. STEMIN’s interactome prefers recruitment by ETS factors, and CTCF, SP1, RBPJ, NFAT5, and TEAD1. In addition, we found many new YAP5SA cofactor associations with DNA binding cofactors ETS1, SP2, SP1, JUNB, FOS, CTGF, IRF3, MEF2C, and RBPJ, as well as its well-known cofactors RUNX1, SMAD3, and TEAD1 [3]. YAP5SA interactomes also revealed considerable association with SRF and its cofactors, previously not shown. Thus, STEMIN and YAP5SA share interactive associations with many more transcription factors than previously imagined, providing a powerful spectrum of transcription regulators that are strongly pro-replicative.

ATAC-seq also revealed chromatin remodeling of many growth factors and signaling pathway genes, including *FGFs*, *BMPs*, *Notchs*, and *Wnts.* [3] Activation of non-canonical WNT5A/B and WNT11, stimulates cardiomyogenic proliferation [59–61]. WNT5A/WNT11 inhibits CTNNB1 signaling and promotes cardiac progenitor development in differentiating embryonic stem cells.^.^ Signaling pathways that express STATS and JAKS, such as STAT5 and JAK3, have key roles in cellular growth [62]. In addition, YAP signaling has a strong impact on inducing IGF1, IGF2, and their binding proteins and gene remodeling to enhance cell growth and resist apoptosis [63].

## Co-expression of STEMIN and YAP5SA repaired infarcted adult mouse hearts *in vivo*

We tested the treatment combination of STEMIN and YAP5SA mRNA *in vivo* by injecting directly into the left ventricles of adult mice after myocardial infarction [4]. The mmRNA injection method with the co-transfectant agent, Lipofectamine MessengerMAX, delivered STEMIN and YAP5SA mmRNA together into 5 precise injection sites surrounding the infarct in the mouse left ventricle proved to be an effective, precise, and leak-free method. In the short-term experiments, we were able to detect incorporated 5-ethynyl-2′-deoxyuridine (alpha-EdU) into DNA of transfected myocytes, which co-stained with anti-SRF and anti-YAP antibodies, around the needle tracts in the mRNA treatment groups. Co-staining with Tnnt and pH3 antibodies marked replicated cardiac myocyte nuclei in response to STEMIN and YAP5SA mRNA injection [4]. Bioinformatic analysis revealed the upregulation of multiple cell cycle gene clusters with co-expression of STEMIN and YAP5SA, while gene clusters associated with cardiomyocyte differentiation (GO: 0055007), sarcomeric assembly and cardiac muscle contraction (GO: 0060048) were profoundly down regulated. We further illustrated the improvement in mouse cardiac function in long-term experiments for 4 weeks. Mice cardiac function evaluated by echocardiography, revealed improved cardiac pumping function by STEMIN and YAP5SA mRNA co-injection.

## STEMIN and YAP5SA may block cardiac apoptosis

Induced myocyte proliferation may not be the only program responsible for the maintenance and or growth of cardiac mass; could the concomitant STEMIN and YAP5SA-induced upregulation of pro-survival and anti-apoptotic miRNAs, as observed from our ATAC-sequencing data [3], be responsible? Preliminary studies revealed transfected STEMIN and YAP5SA mRNAs alone and or in combination in cardiac myocytes for 24 h significantly inhibited CASP3 transcripts by over 65%–90% and inhibited TP53 transcripts primarily with YAP5SA by over 50% (study in preparation). Thus, chromatin remodeling data directed us to hypothesize that the inhibition of cell death may also come into play in the viability of the cardiomyocytes. Studies are underway to determine whether STEMIN and YAP5SA might induce anti-apoptotic miRs through the induction of OKSM.

## Conclusion

Finally, synthetic mRNA may be used as a safe and efficient gene delivery vehicle in adult hearts. Compared to viral vectors, the transient gene expression that mmRNA provides is far more controllable, which makes the mmRNA gene-delivery method a safer option to deliver therapeutic factors for cardiac regeneration. In fact, adenovirus delivery of stem cell factors is initially curative for regenerating cardiac function, but it causes cardiac rhadomyosarcomas in the long term [64]. Given the post-transcription nature of mRNA, mmRNA does not require transfer to the nucleus to get the expression of the target protein. Besides, mmRNA-based gene delivery can deliver gene combinations with different ratios specifically tailored to patients with a different course of the disease. Our data suggest that synthetic mmRNA may be used to deliver STEMIN and YAP5SA into adult cardiac myocytes both *in vitro* and *in vivo* to achieve high transfection efficiency with little biosafety concern. Inducing tissue regeneration by short-term treatments with STEMIN and YAP5SA mRNA may become a useful and safer strategy to treat debilitating human cardiac diseases.
